# The size of the prize for earlier diagnosis of cancer in England

**DOI:** 10.1038/sj.bjc.6605402

**Published:** 2009-12-03

**Authors:** M A Richards

**Affiliations:** 1 National Cancer Action Team, St Thomas’ Hospital, London SE1 7EH, UK

**Keywords:** late diagnosis, avoidable deaths, breast cancer, colorectal cancer, lung cancer

## Abstract

**Background:**

This supplement presents a wide range of observations, reviews, novel research and analyses underpinning the National Awareness and Early Diagnosis Initiative (NAEDI). The preceding three papers present and discuss different aspects of the data from European cancer survival comparison studies. I conclude here by attempting to quantify the extent to which delayed diagnosis in England accounts for observed survival differences and by outlining areas for further research.

**Methods:**

Analysis of indirect evidence related to late diagnosis, surgical intervention rates and utilisation of radiotherapy and chemotherapy in England and other European countries in the late 1990s for breast, colorectal and lung cancer.

**Results:**

Late diagnosis was almost certainly a major contributor to poor survival in England for all three cancers. Low surgical intervention rates are very likely to have contributed to low survival rates for lung cancer and possibly for the other two cancers. Any differences in the use of radiotherapy or chemotherapy are likely to have had only a minor impact on survival differences.

**Conclusion:**

Between 5000 and 10000 deaths within 5 years of diagnosis could be avoided every year in England if efforts to promote earlier diagnosis and appropriate primary surgical treatment are successful. Detailed international benchmarking studies are to be recommended.

In an accompanying paper in this supplement of the *British Journal of Cancer*, Abdel-Rahman *et al* (2009) have estimated the numbers of cancer deaths that would have been avoided each year in Britain (England, Wales and Scotland) if survival had matched that observed elsewhere in Europe. For patients diagnosed over the 15-year period (1985–1999), around 6000–7000 deaths a year that occurred within 5 years of diagnosis would have been avoided if survival in Britain had matched the mean for Europe.

If survival in Britain had matched the best in Europe, around 13 000 premature cancer deaths (those within 5 years of diagnosis) would have been avoided each year among patients diagnosed in the 10 years between 1985 and 1994 and around 11 500 pa for those diagnosed in the years 1995–1999. It is important to note that the ‘highest’ European survival rate was defined conservatively on the basis of the average of the three countries with highest survival rates, excluding Austria and Switzerland at whose data criticisms have been levelled.

The analysis by Abdel-Rahman *et al* (2009) does not, however, attempt to provide estimates of the proportions of these avoidable deaths that may be attributed to late diagnosis, inferior treatment or other factors. Building on the study by Abdel-Rahman *et al* (2009) and the analyses presented by Thomson and Forman (2009) and Møller *et al* (2009), I explore the contributions of different factors to the reported numbers of avoidable deaths for the three cancers with the highest UK mortality and suggest further action and research that might enable us to elucidate this further.

Breast, colorectal and lung cancer accounted for nearly half of the overall numbers of deaths that could have been avoided each year if survival had matched the highest in Europe for cancers diagnosed between 1995 and 1999 (breast cancer – around 2000 pa; colorectal cancer – around 1700 pa and lung cancer – around 1300 pa). Prostate cancer has been excluded from the current analysis as it is highly likely that differences in reported 5-year survival rates relate to differences in prostate-specific antigen testing rates. Avoidable deaths within 5 years of diagnosis may largely reflect a lead-time effect.

## Potential factors accounting for survival differences

Several factors could potentially contribute to international variations in survival rates. These include the following:

- Longer delays leading to more advanced stage of disease at diagnosis in some countries.
- Differences in uptake of screening opportunities for some cancers.
- Access to and quality of primary and adjuvant treatments for cancer.
- Differences in prevalence of co-morbidities between countries.
- Differences in the biology of cancer between countries.

Unfortunately, direct international comparative data are currently unavailable for most of these factors (the possible exception being screening). However, in considering possible explanations for the worse survival rates observed in the United Kingdom, different strands of indirect evidence can be used. First, there are positive indicators of delay and advanced stage contributing to poor survival. Poor 1-year survival rates are generally taken to be an indicator of more advanced disease at diagnosis, though differences in co-morbidities or biology could also contribute. Second, the likely magnitude of the contribution of other factors (e.g. access to and quality of primary treatments) can be explored. In the following sections, I consider the indirect evidence relating to breast, colorectal and lung cancer.

## Breast cancer

There are no studies, which directly compare the extent of delays in diagnosis of breast cancer in the United Kingdom with those in other countries. However, there is evidence that patients in the United Kingdom tend to present with more advanced disease (Sant *et al*, 2003). There is also strong evidence for breast cancer that longer delays are associated with more advanced disease and with poorer survival (Richards *et al*, 1999). Women whose overall duration of symptoms is between 3 and 6 months are estimated to have a 7% lower 5-year survival rate than those with shorter duration of symptoms (Richards *et al*, 1999).

The study by Sant *et al* (2003) involved two cancer registries in the United Kingdom (Thames and Mersey) and related to patients diagnosed in 1990–1992. The proportion of patients presenting with metastatic disease was considerably higher in the Thames Cancer Registry region (10.6%) than in the overall study group (6.2%), which involved registries in Italy, Spain, France, Estonia and the Netherlands. The proportion of patients undergoing surgery was also lower in Thames (83%) and Mersey (88%) than for the group as a whole (90%). Multiple regression analyses of relative survival showed that adjustment for disease stage and surgery greatly reduced the excess risk observed in the two UK registries. The authors concluded that a major reason for the comparatively low survival of breast cancer patients in England was more advanced stage at diagnosis.

Further indirect evidence that advanced stage at diagnosis is a major explanatory factor for the poor survival rates in England comes from the EUROCARE 4 study. As described by Thomson and Forman (2009), 1-year survival rates for women with breast cancer in the United Kingdom were significantly below the European average (91.8 *vs* 93.8%), whereas 5-year survival conditional on surviving 1 year (5∣1 survival) was similar to the average (84.2 *vs* 84.6%).

Surgery remains the mainstay of curative treatment for breast cancer. Patients presenting with inoperable disease have a much worse prognosis than those with operable disease. However, among patients receiving breast surgery, the extent of surgery and the completeness of tumour excision have a relatively minor impact on 5-year survival rates. In addition to this, there is no clear evidence that the quality of breast surgery in the United Kingdom was inferior to that in other European countries at the time of the EUROCARE 4 study (1995–1999). However, a recent study from the Eastern Region of England has raised concerns as to whether all women with breast cancer who might benefit from surgery are receiving it (Wishart *et al*, 2009). In that study, 27% of women over the age of 70 years diagnosed between 1999 and 2003 did not undergo surgery. This figure excluded patients with stage 4 disease.

Radiotherapy is widely used as an adjunct to breast surgery both in women undergoing breast conserving operations and those who are at high risk of local recurrence after mastectomy. Overviews of randomised trials of the impact of radiotherapy (given in addition to breast conserving surgery or mastectomy) have shown highly significant reductions in local recurrence rates and moderate effects on long-term (15 years) breast cancer mortality. Some effect on mortality related to the use of radiotherapy is evident within 5 years of diagnosis (EBCTCG, 2005a; Table 1).

The large majority of women undergoing breast conserving surgery in the United Kingdom in the late 1990s are likely to have received radiotherapy, although low radiotherapy capacity may have led to delays in starting treatment. However, if substantial numbers of women with node-positive disease undergoing breast conserving surgery did not receive radiotherapy, this could also have contributed to poor survival for this group. The impact of any such undertreatment can be estimated from the effect size observed in the randomised trials. For example, if 25% of node-positive patients treated with breast conserving surgery did not receive radiotherapy (which is likely to be a significant overestimate), the overall impact on 5-year survival rates would have been <0.5%, as these patients only constitute a minority of all patients with breast cancer. Some women undergoing mastectomy will also have received radiotherapy to the chest wall, though comparative figures for the United Kingdom and Europe are not available. However, given the modest overall impact of radiotherapy after mastectomy on 5-year survival rates observed in randomised controlled trials, any differences between the United Kingdom and Europe are likely at most to have made only a small contribution to the observed differences in 5-year survival rates and thus to avoidable deaths within 5 years of diagnosis.

Adjuvant chemotherapy and hormonal therapy for early breast cancer undoubtedly reduce local recurrence rates and enhance long-term survival rates (EBCTCG, 2005b). However, the overall impact of adjuvant chemotherapy on 5-year survival rates for women with breast cancer has been estimated to be around 1.5% (Morgan *et al*, 2004). Thus, any differences in utilisation of adjuvant chemotherapy between the United Kingdom and Europe in the period 1995–1999 can only have had a very minor impact on 5-year survival rates – and there is no firm evidence of lower utilisation in the United Kingdom.

The impact of hormonal therapy on 5-year survival rates in randomised trials is significant (Table 1). Tamoxifen will have been the key adjuvant hormonal treatment in the late 1990s both in the United Kingdom and in Europe. In general, uptake of hormonal therapies in the United Kingdom, and Tamoxifen in particular, has been high over the past two decades. It is, therefore, very unlikely that differences in utilisation of hormonal therapies will have accounted for observed differences in survival rates.

Taking all these factors together, there is good evidence that more advanced stage of breast cancer at diagnosis will have been a major contributor to avoidable deaths, partly mediated by lower proportions of patients presenting with operable disease. Underutilisation of surgery in older patients with potentially operable disease may also have been a factor. Differences in the quality of surgery or in utilisation of radiotherapy or adjuvant chemotherapy cannot be excluded, but are likely to be minor contributors. There is no evidence that underutilisation of hormonal therapies was a factor in the United Kingdom. The relatively high uptake of screening in the United Kingdom in the late 1990s compared with that in other European countries should have led to higher survival rates and, therefore, reduced the estimates of avoidable deaths. This further suggests that the problems lie among the large majority of breast cancer patients who presented symptomatically (around 80% in the late 1990s in the United Kingdom).

## Colorectal cancer

As with breast cancer, there is strong evidence that extent of disease at diagnosis (stage) is a major prognostic factor in patients with colorectal cancer. Presentation to hospital as an emergency also carries an adverse prognosis. However, unlike breast cancer, there is no clear relationship between extent of delay, stage and survival.

A EUROCARE high-resolution study has examined factors contributing to variations in survival from colorectal cancer (Gatta *et al*, 2000). This study involved patients diagnosed around 1990 from six European countries (Italy, France, the Netherlands, Spain, United Kingdom and Poland). Two registries in the United Kingdom participated – Mersey and Thames. Across all participating registries, the mean 3-year survival rate was 48%. Survival was lower than average for patients from both of the UK registries (Mersey 44%, Thames 38%). The proportions of patients with Dukes A or B disease were below average in both of the UK registries, and both had a high proportion of deaths within 1 month of diagnosis (Mersey 9%, Thames 16%), even though cases known to the registries through death certificate only or autopsy had been excluded. Cox modelling showed that the outcomes for patients in the United Kingdom were attributable at least in part to unfavourable stage distribution.

Aside from adverse stage distribution, to what extent might differences in practice related to surgery, radiotherapy or chemotherapy account for observed differences in outcomes between Britain and the countries in Europe with the best survival rates? A recent national audit of around two thirds of incident cases of bowel cancer diagnosed in England in 2007 and 2008 showed that 75% underwent some form of surgical treatment, but that only 60% underwent a major resection (Information Centre for Health and Social Care, 2009a). Wide variations in major resection rates were observed between cancer networks. This suggests that a considerable proportion of patients with colorectal cancer in England are presenting either at a stage in which the disease is inoperable or when they are deemed unfit for surgery on other grounds.

The quality of surgical excision is increasingly being recognised as a prognostic factor both in rectal cancer (Quirke *et al*, 2009) and colonic cancer (West *et al*, 2008; Hohenberger *et al*, 2009). Total mesorectal excision of rectal cancer was pioneered in the United Kingdom in the late 1980s (Heald and Ryall, 1986). However, it was adopted into routine clinical practice in some other European countries during the early 1990s (Wibe *et al*, 2002; Martling *et al*, 2005), but not until a decade later in the United Kingdom. This could certainly contribute to observed differences in survival rates for rectal cancer between European countries for patients diagnosed in the late 1990s and the early years of this century.

Radiotherapy (either preoperative or postoperative) has a significant function in the management of rectal cancer, but not of colon cancer. In the Swedish Rectal Cancer Trial (1997), the addition of a short course of pelvic radiotherapy before surgery resulted in a significant improvement in overall survival at 5 years (58 *vs* 48%). However, in the NSABP R-02 study (Wolmark *et al*, 2000), the addition of postoperative radiation therapy to chemotherapy in Dukes B and C rectal cancer had no beneficial effect on survival (though locoregional relapse was reduced). Differences in utilisation of radiotherapy for rectal cancer between European countries could, therefore, potentially contribute to observed differences in 5-year survival rates. However, the extent of any differences in utilisation is difficult to quantify.

Adjuvant chemotherapy has an established function in the management of patients with lymph node-positive (Dukes C) colorectal cancer, but is of uncertain benefit in Dukes B disease. Morgan *et al* (2004) estimated that the use of adjuvant chemotherapy contributes 1.8% to 5-year survival rates for colon cancers in Australia. This was on the basis of a 5% benefit being observed in patients with Dukes C disease (35% of those with colon cancer in Australia), but assumed no benefit in patients with Dukes B disease (Dube *et al*, 1997; International Multicentre Pooled Analysis of Colon Cancer Trials (IMPACT B2) Investigators, 1999). The comparable estimate for the United States was a 1.0% contribution to 5-year survival. The lower figure for the United States is attributable to the smaller proportion of patients presenting with Dukes C disease (21%).

Morgan *et al* (2004) considered that all patients with Dukes B or C rectal cancer would benefit from adjuvant chemotherapy and that the improvement in overall survival with chemotherapy alone would be 9%. As Dukes B and C patients constituted 60% of all rectal cancers in Australia, the benefit across all rectal cancers was 5.4%. The comparable figure for the United States was 3.4%, on the basis of 38% being Dukes B or C disease. The authors acknowledge that the figures for rectal cancer in both countries may be an overestimate as the benefit may only exist for Dukes C cancer.

There are no available figures to show whether utilisation of adjuvant chemotherapy for colorectal cancer was lower in the United Kingdom in the late 1990s than in the European countries with the highest 5-year survival rates, but this must remain a possibility. If utilisation in the United Kingdom had been half that in the countries with the highest survival rates and if the benefits estimated for Australia had been realised in the ‘best’ European countries, this would account for a difference of around 0.9% in 5-year survival rates for colon cancer and 2.7% for rectal cancer. As colon cancer accounts for approximately two thirds of colorectal cancers, the combined figure would be around 1.5%.

## Lung cancer

As with breast and colorectal cancer, stage of disease at diagnosis has a major impact on 5-year survival rates (Mountain, 1997). Although there have been no direct international comparisons of extent of delays or of stage at diagnosis for lung cancer, delays have been reported from a variety of countries (Birring and Peake, 2005) and the finding that 1-year survival rates in England (26.9%) was well below the European average (36.0%) in the EUROCARE 4 study suggests that late diagnosis is a major factor.

The large majority of lung cancer patients in all countries present with inoperable disease. However, for those whose disease is amenable to surgery, 5-year survival rates are reasonably good (Mountain, 1997). Presenting with operable disease and having access to surgery are, therefore, of very high prognostic importance in lung cancer.

Although there are no direct international comparisons of lung cancer resection rates, these have been reported for individual countries (Table 2). Resection rates in Rotterdam (the Netherlands), Sweden, Varese (Italy) and the United States all seem to be considerably higher than those recently reported from England and Wales. The figures for England and Wales come from the National Lung Cancer Audit (the LUCADA database), which includes approximately 75% of incident cases in these countries (Information Centre for Health and Social Care, 2009b). They are considered to be representative of surgical activity in the country as a whole. It is noteworthy that within England and Wales, there is wide variation in resection rates between cancer networks (regions typically covering populations of 1–2.5 million).

The surgery involved in lung cancer is significantly more invasive and technically complex than, for example, that required for breast cancer and patient fitness, age and co-morbidities together with the availability of specialist surgery that are important factors in decision making and resection rates. As the median age at diagnosis is around 71 years in the United Kingdom (Information Centre for Health and Social Care, 2009b) and the majority of lung cancer patients are smokers or ex-smokers, their rates of cardio-respiratory co-morbidities are high. These high co-morbidity rates are likely to apply to lung cancer patients in all countries, though there is some evidence that co-morbidity rates are higher among lung cancer patients in the United Kingdom (Imperatori *et al*, 2005). It is currently impossible to assess the relative contribution of late diagnosis, patient fitness and poor access to specialist surgery to the low resection rates in the United Kingdom. However, it is likely that failure to identify patients who might be suitable for surgery has been at least part of the problem. A report from Leicester in England showed that resection rates doubled after the establishment of a multidisciplinary team and the appointment of a specialist thoracic surgeon (Martin-Ucar *et al*, 2004).

To what extent are differences in the use of chemotherapy for lung cancer between the United Kingdom and the countries in Europe with the highest survival rates likely to impact on 5-year survival rates? From the National Lung Cancer Audit, we know that only 62% of patients with small cell lung cancer (SCLC) were reported as receiving chemotherapy and it is very likely that this proportion is significantly higher elsewhere in Europe. However, 5-year survival from SCLC is very low in all countries. Among patients with non-small cell lung cancer, utilisation of adjuvant chemotherapy will undoubtedly be lower than that in countries with high resection rates. However, the number of patients benefiting would be small. It might, therefore, be reasonable to estimate that the gap in 5-year survival rates between the United Kingdom and the best in Europe would be narrowed by 0.5–1.0%, if chemotherapy utilisation was the same. This would, in turn, require more patients to be diagnosed at an operable stage and be referred for surgery.

## Discussion

For breast, colorectal and lung cancers, 1-year survival in England is below the European average and considerably below that observed in countries with the highest survival in Europe. For breast and colorectal cancer, there is good evidence, albeit relating to the early 1990s, that the poor 5-year survival observed in England was at least in part attributable to patients having more advanced disease at diagnosis. For lung cancer there is increasing evidence, as outlined above, that fewer patients in England (and Wales) undergo lung resection than in other countries. It seems likely that this is due to a combination of late diagnosis and failure to identify and refer patients who are suitable for surgery. Low rates of surgical intervention for breast and colorectal cancer may also contribute to poor survival rates, though comparative data are currently lacking.

Chemotherapy given to patients presenting with metastatic breast, colorectal or lung cancer is likely to have had only a marginal impact on 5-year survival rates for each cancer, as survival is poor whether or not a patient receives chemotherapy. Differential use of adjuvant chemotherapy for each cancer type between countries could contribute to the overall gap in survival rates. However, from the estimates made by Morgan *et al* (2004), the overall contribution of chemotherapy to 5-year survival seems to be relatively small. Any differences because of variations in utilisation of adjuvant chemotherapy will inevitably be smaller. Similarly, differences in utilisation of radiotherapy are likely to have only a modest impact on 5-year survival.

In conclusion, late diagnosis and low surgical intervention rates seem to be by far the most important factors underlying the poor 5-year survival rates observed in England. Although exact estimates are impossible, it seems highly likely that these factors accounted for the large majority of the avoidable deaths in England observed during the 1990s for patients with breast, colorectal and lung cancer. There is no reason to think that the same factors will not apply in some, if not all, other cancers for which survival in England is below the European average. It is, therefore, likely that most (i.e. between 5000 and 10 000) of the avoidable deaths observed each year in the late 1990s when assessed against the highest survival in Europe were attributable to late diagnosis, combined with low utilisation of potentially curative treatments.

How are things likely to have changed over the past decade? One-year survival rates have continued to improve in England for breast cancer and colorectal cancer and are likely to have improved in other countries too. Little change has been observed in 1-year survival for lung cancer. Major progress has been made over the past decade in England in relation to cutting hospital waiting times, establishing specialist multidisciplinary teams and reconfiguring complex surgical services. Screening for breast cancer has been extended and improved and screening for bowel cancer has been introduced. However, until very recently much less attention has been paid to the problem of late diagnosis. Given the findings outlined in this paper, it is, therefore, likely that the gap in 5-year survival will have remained substantial.

Efforts now need to be directed at promoting early diagnosis for the very large number (over 90%) of cancer patients who are diagnosed as a result of their symptoms, rather than by screening. National Awareness and Early Diagnosis Initiative has been established to coordinate and drive efforts in this area. The size of the prize is large – potentially 5000 to 10 000 deaths that occur within 5 years of diagnosis could be avoided every year.

This analysis has highlighted the lack of direct comparative data enabling definitive statements to be made on the reasons for survival differences between countries and on the numbers of lives that might be saved through different interventions. Detailed international benchmarking studies are now needed to explore the contributions of the factors considered in this paper. Alongside, up-to-date comparisons of survival, high-resolution studies are needed to examine potential differences in stage, co-morbidity, biology and treatments. It would also be valuable to measure delays in different countries and the factors, which may contribute to delays. In addition to these benchmarking studies, further primary research should be encouraged to assess the effectiveness and cost effectiveness of interventions to promote earlier presentation.

## Conflict of interest

The author declares no conflict of interest.

## Figures and Tables

**Table 1:** Impact of adjuvant therapies on 5-year breast cancer mortality

	**5-year breast cancer mortality**		
	**Without (%)**	**With (%)**	**Difference (%)**
*Radiotherapy*			
Breast-conserving surgery (node negative)	8.9	8.0	0.9
Breast-conserving surgery (node positive)	24.3	20.9	3.4
Mastectomy+axillary clearance (no)	12.5	11.3	1.2
Mastectomy+axillary clearance (n+)	34.0	32.1	1.9
			
*Chemotherapy*			
Polychemotherapy (age <50 years)	20.4	15.7	4.7
Polychemotherapy (age 50–69 years)	21.3	18.7	1.6
			
*Hormonal therapy*			
Tamoxifen (about 5 years) in ER-positive or ER-unknown disease	11.9	8.3	3.6
Ovarian ablation or suppression in ER-positive or ER-unknown disease	18.4	16.6	1.8

Abbreviations: ER=estrogen receptor.

*Note*: Figures taken from EBCTCG (2005a, 2005b).

**Table 2:** Lung cancer resection rates

**Reference**	**Location**	**Period**	**Resection rate (%)**
Damhuiss and Schutte (1996)	Rotterdam (Netherlands)	1984–1992	20
Myrdal *et al* (2009)	Sweden	1995–2003	17.5
Fry *et al* (1999)	USA	1985–1995	27^*^
Imperatori *et al* (2005)	Varese (Italy)	?	25
Information Centre for Health and Social Care, 2009b	England +	2005–2007	9–10.2
	Wales		12

^*^These figures relate to patients for whom a histological diagnosis had been made, rather than to the entire population of lung cancer patients.
